# An Immunoinformatics Study to Predict Epitopes in the Envelope Protein of SARS-CoV-2

**DOI:** 10.1155/2020/7079356

**Published:** 2020-11-25

**Authors:** Renu Jakhar, S. K. Gakhar

**Affiliations:** Centre for Medical Biotechnology, Maharshi Dayanand University, Rohtak 124001, Haryana, India

## Abstract

COVID-19 is a new viral emergent disease caused by a novel strain of coronavirus. This virus has caused a huge problem in the world as millions of people are affected by this disease. We aimed at designing a peptide vaccine for COVID-19 particularly for the envelope protein using computational methods to predict epitopes inducing the immune system. The envelope protein sequence of SARS-CoV-2 has been retrieved from the NCBI database. The bioinformatics analysis was carried out by using the Immune Epitope Database (IEDB) to predict B- and T-cell epitopes. The predicted HTL and CTL epitopes were docked with HLA alleles and binding energies were evaluated. The allergenicity of predicted epitopes was analyzed, the conservancy analysis was performed, and the population coverage was determined throughout the world. Some overlapped CTL, HTL, and B-cell epitopes were suggested to become a universal candidate for peptide-based vaccine against COVID-19. This vaccine peptide could simultaneously elicit humoral and cell-mediated immune responses. We hope to confirm our findings by adding complementary steps of both *in vitro* and *in vivo* studies to support this new universal predicted candidate.

## 1. Introduction

As we all know, the coronavirus has stopped the movements of the entire world. This virus is so deadly that it is taking lives of the more than thousands of people every day and affecting millions of people on the globe. However, the disease was first reported in the Wuhan city of China, where the virus was isolated from a patient with respiratory symptoms in December 2019 [1, 2], later identified it by the name of COVID-19 [3]. The World Health Organization (WHO) announced this disease as a pandemic disease that spread from China to more than a hundred countries in the world. The disease had already struck more than million persons of whom thousands of peoples died from COVID-19 infection and the majority of them were reported from China, Italy, the United State of America, Britain, and Spain.

Coronaviruses are the large group of viruses belonging to the family Coronaviridae and the order Nidovirales that are common among animals [4]. The Coronaviridae family is divided into four genera based on their genetic properties, including alpha, beta, gamma, and delta coronavirus genus [5]. The 2019-nCoV is an enveloped positive-sense RNA, beta coronavirus with a genome of 29.9 kb [6]. They are zoonotic, transmitted from animals to humans [7]. COVID-19 affects the respiratory system (lungs and breathing tubes). Most COVID-19 patients developed severe acute respiratory illness with symptoms of fever, cough, and shortness of breath. Maximum reported cases of COVID-19 have been linked through travel to or residence in countries in this region [8, 9].

Presently, there are no clinically approved vaccines available in the world for this disease. The development of a new vaccine for this new emergent strain by using therapeutic and preventive approach can be readily applied to save human lives. The use of peptides or epitopes as therapeutics is a good strategy [10] as it has advances in design, stability, and delivery [11, 12]. Moreover, there is a growing importance on the use of peptides in vaccine design by predicting immunogenic CTL, HTL, and B-cell epitopes from tissue-specific proteins of organisms [13, 14]. Among the structural proteins of SARS-CoV-2, the CoV envelope (E) protein is a small integral membrane protein involved in several aspects of the life cycle of the virus, such as envelope formation, assembly, budding, and pathogenesis [15]. Thus, it is considered to be a promising target for effective COVID-19 vaccine design. More importantly, T-cell-based cellular immunity is essential for cleaning SARS-CoV-2 infection because it is memory based [16, 17]. The E protein is a highly conserved protein having very low mutation rate. This protein can elicit both cellular immunity, and neutralizing antibody against COVID-19 is necessary for efficient vaccine development [18, 19].

Therefore, in this study, an immunoinformatics-based approach was adopted to identify candidate epitopes against the envelope protein of SARS-CoV-2 that could appropriately trigger significant cellular and humoral immune responses [20, 21]. The aim of this study is to analyze envelope protein strains using *in silico* approaches looking for the conservancy, which is further studied to predict all potential epitopes that can be used after *in vitro* and *in vivo* confirmation as a candidate for therapeutic peptide vaccine [22–24] and as to be used as a diagnostic screening test.

## 2. Materials and Methods

### 2.1. Protein Sequence Retrieval

The protein sequence of envelope protein from severe acute respiratory syndrome coronavirus 2 isolate Indian strain (SARS-CoV-2/166/human/2020/IND) with accession no. QIA98585.1 was retrieved from the NCBI database. The antigenicity of this sequence was predicted by the VaxiJen v2.0 server [25] with the default parameter. VaxiJen predicts a protein as an antigenic protein if the score is above the threshold. Also, E protein sequences were isolated from different coronavirus species. Further, the multiple sequence alignment of envelope protein sequences was carried out through Clustal W. Also, the E protein sequences of SARS-Co-V were retrieved from the NCBI database from different parts of the world till date (09/09/20); retrieved sequences and their accession numbers are listed in the supplementary file.

### 2.2. Homology Modelling

The 3D structure of the envelope protein was obtained by SWISS-MODEL which uses homology detection methods to build 3D models [26]. UCSF Chimera was used to visualize and minimize the 3D structures [27], and structure validation was carried out with SAVES [28]. Homology modelling was achieved to establish conformational B-cell epitope prediction and for further verification of the surface accessibility and hydrophilicity of B-lymphocyte epitopes predicted, as well as to visualize all predicted T-cell epitopes at the structural level.

### 2.3. B-Cell Epitope Prediction

B-cell epitope is the portion of an immunogen, which interacts with B-lymphocytes. As a result, the B-lymphocyte is differentiated into an antibody-secreting plasma cell and the memory cell. Thus, the IEDB resource was used for analysis. Envelope protein was subjected to Bepipred linear epitope prediction [29], Emini surface accessibility [30], Kolaskar and Tongaonkar antigenicity [31], Parker hydrophilicity [32], Chou and Fasman beta turn [33], and Karplus and Schulz flexibility prediction [34] prediction methods in IEDB that predict the probability of specific regions in the protein to bind to B-cell receptor, being in the surface, being immunogenic, being in a hydrophilic region, and being in a beta turn region, respectively. Potentially continuous B-cell epitope was predicted using tool Ellipro from IEDB resource [35].

### 2.4. Conservancy, Allergenicity, and Toxicity Analysis of Epitopes

The conserved epitope analysis was carried out in the E protein sequences of SARS-CoV-2 from different parts of the world by analysing conservation across antigens using IEDB. The allergenicity of predicted epitopes was analyzed by AllerTOP tool [36]. ToxinPred server was used to predict the toxicity assessment of epitopes [37].

### 2.5. Prediction of Cytotoxic T-Cell Epitope

T-cell epitopes were predicted by the NetCTL server [38]. The parameter was set at 50 to have the highest specificity and sensitivity of 0.94 and 0.89, respectively, and all the supertypes were taken during the submission of a protein sequence. A combined algorithm of major histocompatibility complex-1 (MHC-1) binding, transporter of antigenic peptide (TAP) transport efficiency, and proteasomal cleavage efficiency were used to predict the overall scores [39]. On the basis of the combined score, first five best epitopes were selected for further testing as putative epitope vaccine candidates. MHC-1 binding T-cell epitope was predicted by IEDB by using the stabilized matrix method (SMM) for each peptide [40]. Prior to prediction, all epitope lengths were set as 9mers, and conserved epitopes that bind to many HLA alleles at score equal or less than 1.0 percentile rank were selected. IC50 below 200 nM shows maximum interaction potentials of CTL epitope and MHC-I allele. For further analysis, alleles having IC50 less than 200 nm were selected. Overall, CTL epitopes having the higher immunogenicity are selected than those having lower immunogenicity. Therefore, the IEDB immunogenicity prediction tool was used for the prediction of the immunogenicity of the candidate epitopes [41].

### 2.6. Prediction of Helper T-Cell Epitope

Analysis of peptide binding to MHC class II molecules was assessed by the IEDB MHC-II prediction tool, where the SMM-based NetMHCIIpan 3.0 server was used [42]. It covers all HLA class II alleles including HLA-DR, HLA-DQ, and HLA-DP [35]. The IEDB recommends a consensus method to make selections based on a percentile rank of the top 10%. Alternatively, NetMHCIIpan 3.0 selects peptides based on binding affinity. Epitopes with low IC50 are good binders. IC50 below 200 nM shows maximum interaction potentials of HTL epitope and MHC-II allele. So epitopes with binding affinity to alleles with IC50 less than 200 nm with lower percentile score are selected [43]. Accordingly, five top epitopes were selected. The predicted HTL epitopes were submitted to the IFN epitope server to check whether the MHC-II binding epitopes had the ability to induce IFN-*γ* [44].

### 2.7. Population Coverage Calculation

All potential MHC-I and MHC-II binders from envelope protein were assessed for population coverage against the whole world population that had been reported COVID-19 cases. Calculations were made using the selected MHC-I and MHC-II interacted alleles by the IEDB population coverage calculation tool [45].

### 2.8. Docking Studies

Epitopes of MHC-I and MHC-II alleles that were predicted to bind with higher affinity and have percentile rank below 1.0 were selected as the ligands, which are modelled using PEP-FOLD online peptide modelling tool [46]. The receptor MHC-I and MHC-II alleles' 3D structure was obtained from the PDB server [47]. PatchDock program was used for all dockings [48]. CHIMERA and Ligplot were used for visualization and determination of binding affinity and to show the suitable epitope binding residues with HLA.

## 3. Results

### 3.1. Retrieval of Protein Sequence and Antigenicity Determination

**Figure 1:**
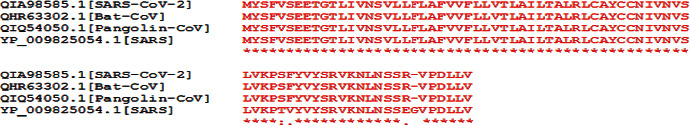
Alignment of the amino acid sequences of the E protein of four coronaviruses along with their accession no.

The protein sequence of the envelope protein (Accession no. QIA98585.1) from severe acute respiratory syndrome coronavirus 2 isolate Indian strain was retrieved in FASTA format. The VaxiJen server used for antigenicity prediction uses a threshold for assessment of antigenicity. This protein was predicted to be an antigenic protein with an overall score of 0.6 which is higher than the threshold score (0.4). The envelope protein sequences retrieved from the NCBI database from different areas were aligned, to see the conservation of protein. Also, coronavirus sequences retrieved from the NCBI database were aligned, and the conserved regions of E protein were selected for epitope prediction (Figure 1).

Primary structure analysis revealed that the envelope glycoprotein of SARS-CoV-2 had a molecular weight of 8365 D with 75 aa length. The theoretical isoelectric point (pI) is 8.57. An isoelectric point above 7 indicates a positively charged protein. The instability index (II) was computed to be 38.6. This categorizes the protein as stable. The aliphatic index of 144indicates that it is thermostable in nature. The positive grand average of hydropathicity (GRAVY) of 1.128 indicates that is hydrophobic in nature. The amino acid Val (V) and Leu (L) were found in rich amounts in the protein. The TMHMM online server showed that residues 1–11 were presented inside region, residues 12–34 were within the transmembrane, and residues 35–75 were outside the region of the protein.

### 3.2. Homology Modelling, Refinement, and Validation of E Protein

**Figure 2:**
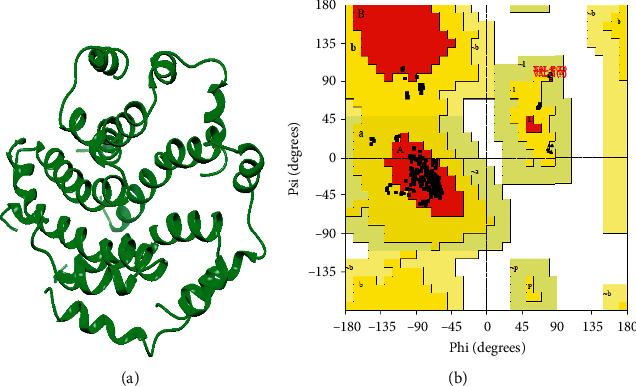
(a) Predicted 3D structure of putative E protein by SWISS-MODEL. (b) Validation of 3D structure of E protein by Ramachandran plot.

The three-dimensional structure of the envelope protein of the SARS-CoV-2 was modelled using the homology structure modelling tool SWISS-MODEL (Figure 2(a)). This protein showed a good model with SWISS-MODEL by using PDB ID: 5X29as a template having 91.3% identity and 54% similarity with the query structure. These models were energy minimized by using Chimera. The Ramachandran plot (Figure 2(b)) indicated that 84.4% residues were in the most favoured region, 14.1% in the additional allowed region, 1.5% in the generously allowed region, and 0% in the disallowed region for the modelled envelope protein.

### 3.3. Prediction of Conformational and Linear B-Cell Epitope

The conformational B-cell epitopes were also obtained in five chains of envelope protein by using ElliPro. ElliPro gives the score to each output epitope, which is protrusion index (PI) value averaged over each epitope residue [49]. Some ellipsoids approximated the tertiary structure of the protein. The highest probability of a conformational epitope was calculated at 76% (PI score: 0.76). Residues involved in conformational epitopes, their number, location, and scores are shown in Table 1. ^60^SRVKNL^65^ residues were found to have highest PI score. This epitope was found to be antigenic, nontoxic, and conserved in all the strains of coronaviruses shown here.

**Figure 3:**
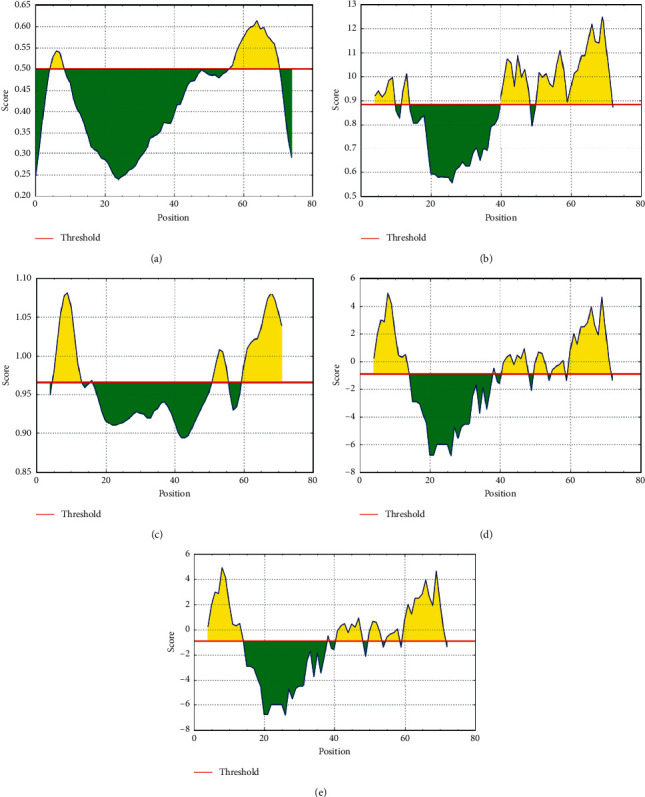
Prediction of B-cell epitopes by different scales/parameters (a–e). Yellow areas above the threshold (red line) are proposed to be a part of B-cell epitope. Epitope SRVKNL from 60–65 position satisfies the threshold values of all the parameters. (a) Bepipred linear epitope prediction. (b) Chou and Fasman beta turn prediction. (c) Karplus and Schulz flexibility prediction (d). Emini surface accessibility prediction. (e) Parker hydrophilicity prediction.

Envelope protein was subjected to Bepipred linear epitope prediction, Emini surface accessibility, Karplus and Schulz flexibility prediction, Parker hydrophilicity, and Chou and Fasman beta turn prediction methods in IEDB that predict the probability of specific regions in the protein to bind to B-cell receptor, being in the surface, being immunogenic, being in a hydrophilic region, and being in a beta turn region, respectively (Figure 3).

In the Bepipred linear epitope prediction method, the average binder score of envelope protein to B cell was 0.421, with a maximum of 0.613 and a minimum of −0.239, and all values equal to or greater than the default threshold 0.023 were predicted to be potential B-cell binders. In Emini surface accessibility prediction, the average surface accessibility areas of the protein were scored as 1.000, with a maximum of 4.316 and a minimum of 0.088, and all values equal to or greater than the default threshold 1.0 were potentially in the surface. The default threshold of antigenicity of the protein was 1.119, with a maximum of 1.262 and a minimum of 0.947. In Parker's hydrophilicity prediction, the average hydrophilicity score of the protein was 1.480, with a maximum of 4.929 and a minimum of −6.843, and all values equal to or greater than the default threshold −0.911 were potentially hydrophilic. The Chou and Fasman beta turn prediction method was used with the default threshold of 0.883 with a maximum of 1.264 and a minimum of 0.883 for more confirmation for the prediction of the epitope to elicit B cell employed. The Karplus and Schulz flexibility prediction method was used with the default threshold of 0.965 with a maximum of 1.081 and a minimum of 0.894 for more confirmation for the prediction of the epitope to elicit B cell employed. Two epitopes ^4^FVSEET^9^ and ^54^PSFYVYSRVKNLNSSRVP^71^ are predicted by Emini surface accessibility and Bepipred linear epitope prediction methods. The predicted conformational B-cell epitope ^60^SRVKNL^65^ was found to satisfy the threshold of all the above said linear B-cell epitope prediction methods and was found to be nonallergic in nature (Figure 3).

### 3.4. Prediction of Cytotoxic T-Lymphocyte Epitopes and Interaction with MHC Class I

Envelope protein from the SARS-CoV-2 was analyzed using the IEDB MHC-I binding prediction tool to predict the T-cell epitope suggested interacting with different types of MHC class I alleles. Based on NetCTL and SMM-based IEDB MHC-I binding prediction tools, epitopes interacted with different MHC-I alleles with higher affinity (IC50 less than 200) were selected. The predicted proteasome score, tap score, MHC score, processing score, and MHC-I binding score are summarized as a total score in Table 2. These epitopes are antigenic and nonallergic. The peptide FLAFVVFLL from 20 to 29 had highest immunogenicity, combined score, and affinity to interact with 5 alleles (HLA-A*∗*02:01, HLA-A*∗*02:06, HLA-B*∗*15:02, HLA-C*∗*03:03, and HLA-A*∗*68:02), followed by FLLVTLAIL from 26 to 34 and LLFLAFVVF from 18 to 26 that had an affinity to interact with 5–6 alleles for each. The epitope VLLFLAFVV had good immunogenicity with good population. The epitopes and their corresponding MHC-I alleles are shown in Table 2.

### 3.5. Prediction of Helper T-Lymphocyte Epitopes and Interaction with MHC Class II

By the same way in IEDB MHC-I binding prediction tool, T-cell epitopes from the SARS-CoV-2 were analyzed using the MHC-II binding prediction method, based on SMM-based NetMHCIIpan with IC50 less than 200. There were top 5 predicted epitopes found to be nonallergic and antigenic for which the peptides (core) FLAFVVFLL and LLVTLAILT had high affinity to interact with nine alleles. Moreover, the FLAFVVFLL epitope was found to have maximum population coverage followed by LLVTLAILT, LAILTALRL, and VLLFLAFVV epitopes. The results are listed in Table 3. These epitopes show positive results, which confirms that they have the capability to induce IFN-*γ*.

There were several overlapping between MHC class I and MHC class II epitopes. The overlapping epitopes are found from amino acid sequences 16 to 34 for MHC class I and II alleles, suggesting the possibility of antigen presentation of this region to immune cells via both MHC class I and II pathways, i.e., ^16^SVLLFLAFV^24^, ^17^VLLFLAFVV^25^, ^18^LLFLAFVVF^26^, and ^26^FLLVTLAIL^34^ (Tables 2 and 3). MHC class II epitopes, ^31^LAILTALRL^39^ and ^57^YVYSRVKNL^65^, are found to have overlapping conformational B-cell epitope (Table 1). An ideal epitope should be highly conserved. The conservancy analysis of these epitopes indicated that all these CTL and HTL epitopes are found to be >99.9% conserved in all sequences of the SARS-CoV-2 considered in this study (Supplementary file).

### 3.6. Analysis of the Population Coverage

**Figure 4:**
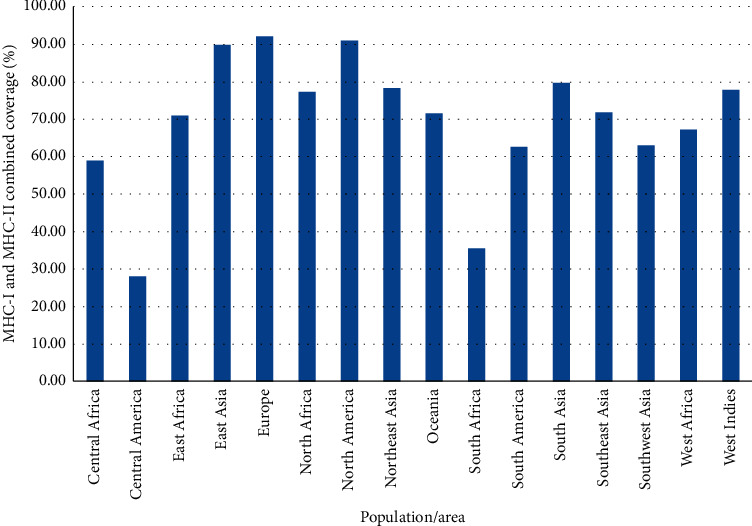
MHC-I and MHC-II epitopes (combined) population coverage among different geographic regions around the world.

Epitopes that are suggested interacting with MHC-I and II alleles (especially high-affinity binding epitopes and that can bind to a different set of alleles) were selected for population coverage analysis. The results of population coverage of all epitopes are listed in Tables 2 and 3. All the epitopes that interact most frequently with MHC class I and II alleles gave a high percentage against the whole world population calculated by the IEDB population coverage tool (Figure 4). The maximum class I and II combined population coverage (92.1%) for these proposed epitopes was found in Europe, while the higher population coverage was found in North America (90.9%) and East Asia (89.8%) followed by South Asia (79.6%) and Northeast Asia (78.9%), West Indies (77.8%), North Africa (77.2%), Southeast Asia and Oceania (71.8%), East Africa (70.8%) and West Africa (67.2), and South America and Southwest Asia (62.9%).

### 3.7. Molecular Docking of MHC-I and MHC-II Alleles with Predicted T-Cell Epitope

The predicted T-cell epitope that interacted with selected human's MHC-I and MHC-II alleles were used as ligands to detect their interaction with alleles/receptors, by docking techniques using online software PatchDock. After successful docking by PatchDock, the refinement and rescoring of the docking results were carried out by the FireDock server. After refinement of the docking scores, the FireDock server generates global energies/binding energies for the best solutions. Chimera and Ligplot were used to visualize the best results. The 3D structure of epitopes was predicted using PEP-FOLD, and energy minimization was carried out by using Chimera. Ten models were built from this tool, and based on the conformation, the best one was selected. Based on the binding energy in kcal/mol unit, the lowest binding energy (kcal/mol) was selected to obtain the best binding (pose) and to predict real CTL and HTL epitope as possible.

**Figure 5:**
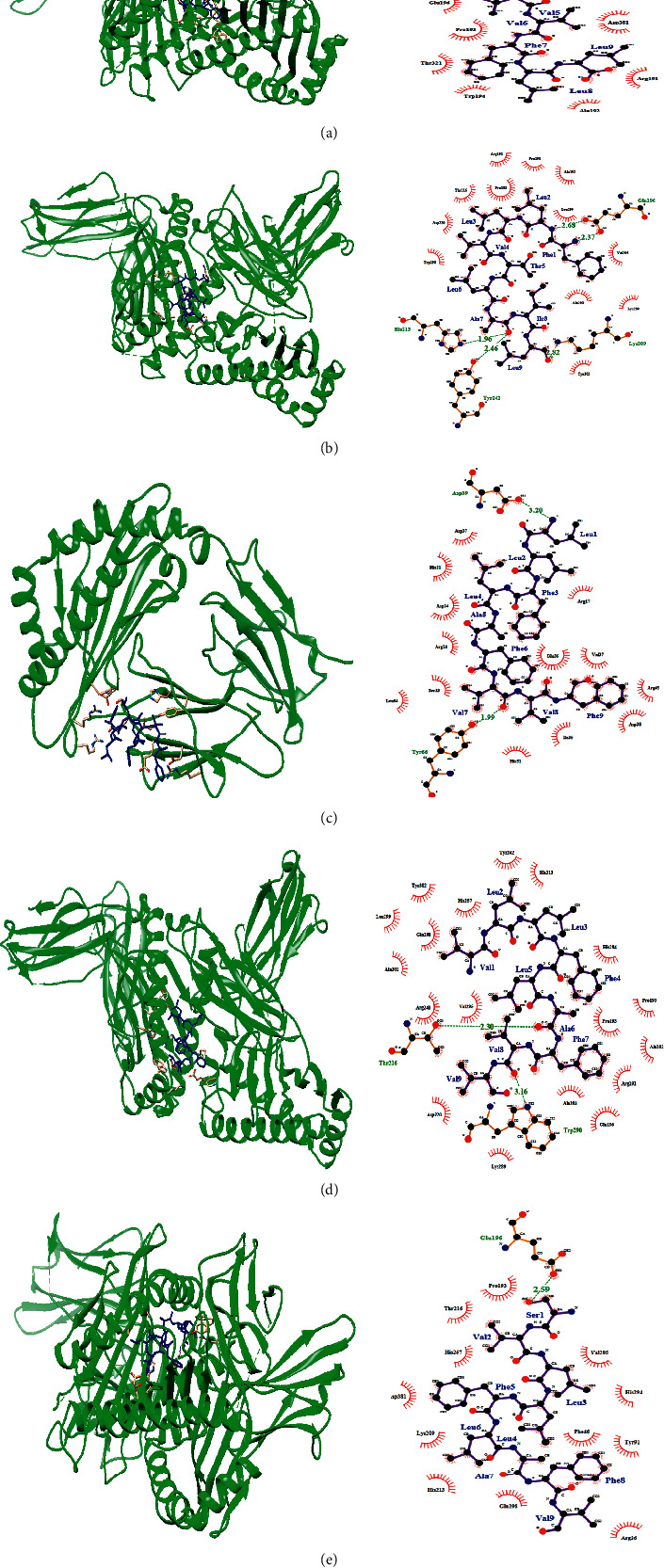
(Left) docking sites of predicted peptide against selected MHC-I (6APN and 1EFX) receptors. HLAs are shown in ribbon (green) form and epitope is shown in stick (blue). (Right) interacting residues between ligand and receptor. H-bond interaction is shown in green colour. (a) FLAFVVFLL-6APN; (b) FLLVTLAIL-6APN; (c) LLFLAFVVF-1EFX; (d) VLLFLAFVV-6APN; (e) SVLLFLAFV-6APN.

**Figure 6:**
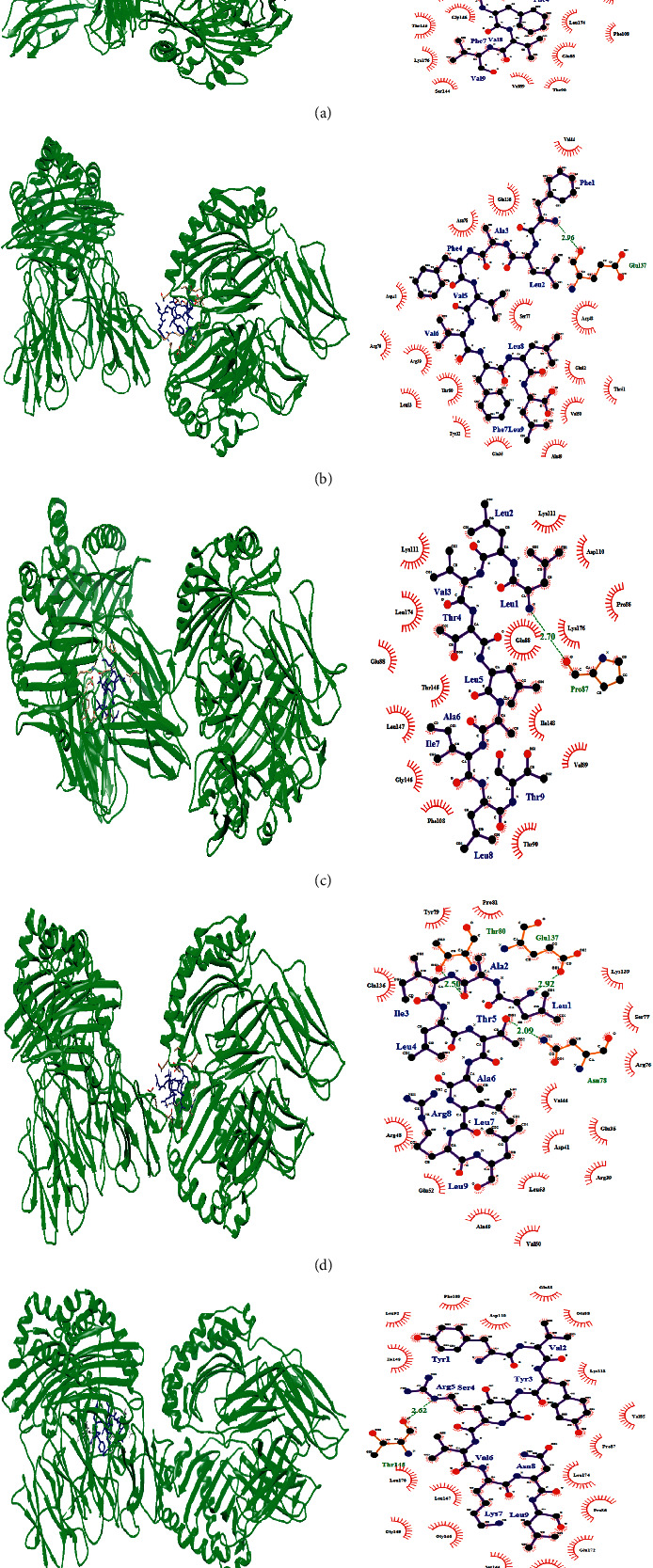
(Left) docking sites of predicted peptide against selected MHC-II (1AQD) receptor. HLAs are shown in ribbon (green) form and epitope is shown in stick (blue). (Right) interacting residues between ligand and receptor. H-bond interaction is shown in green colour. (a) VLLFLAFVV-1AQD; (b) FLAFVVFLL-1AQD; (c) LLVTLAILT-1AQD; (d) LAILTALRL-1AQD; (e) YVYSRVKNL-1AQD.

The receptors used for docking studies included reported HLAs, HLA-A*∗*02:01(PDB ID: 6APN) and HLA-C*∗*03:03 (PDB ID: 1EFX) for class I and HLA-DRB1*∗*01:01 (PDB ID: 1AQD) for class II. VLLFLAFVV was observed to have interaction with the MHC-I (PDB ID: 6APN) and MHC-II (PDB ID: 1AQD) with lowest binding energy, −80.3 kcal/mol and −90.4 kcal/mol, respectively (Figures 5 and 6). Also, HLA-A*∗*02:01 and HLA-DRB1*∗*01:01 were observed to have interaction with the FLAFVVFLL epitope with lower binding energy. The predicted peptide showed significant binding affinities with all HLAs (Figures 5(a) and 6(a)). Similarly, the binding energy was calculated for other MHC-I and MHC-II epitopes and their binding energies were found to be negative (Tables 2 and 3). The residues of epitope interacted with HLAs residues through hydrogen bonding (H-bond). The residues of epitope that contacts the residues of the HLA structure are shown in the right part of Figures 5(b) and 6.

## 4. Discussion

In this study, we aimed to determine the highly potential immunogenic epitopes for B and T cells, the prime molecules of humoral, and cell-mediated immunity as peptide vaccine candidates for COVID-19 infection using the envelope protein as a target. The envelope protein plays an important role in the virion assembly and propagation of virus inside. Sequence alignment of the envelope protein across four strains of coronavirus was done which shows total conservation. Envelope protein is relatively conserved and highly immunogenic as compared to other structural proteins of SARS-CoV-2. Conservancy in E protein in the SARS-CoV-2 was found promising for peptide vaccine design. The physicochemical characteristics of SARS-CoV-2 envelope protein show stable, hydrophobic, and aliphatic nature. We have modelled the three-dimensional structure of the envelope protein of the SARS-CoV-2 using homology structure modelling tool: SWISS-MODEL and the NMR structure of the SARS coronavirus E protein pentameric ion channel with PDB ID: 5X29 as a template. This protein exists in both monomeric and homopentameric forms [17]. The conformational epitopes were predicted by using a 3D structure of the E protein. The B-cell epitope residue, SRVKNL located on the surface of the E protein, had good protrusion index (PI) score (0.76) which were indicative of high accessibility. Ellipsoid value of PI 0.76 indicates that 76% of protein residues lie within ellipsoid and the remaining 24% residues lie outside. PI score and solvent accessibility are directly proportional to each other if the PI score is higher; maximum is the solvent accessibility of the residues [49]. The potential and effective linear B-cell epitope should get above threshold scores in Bepipred linear epitope prediction, Emini surface accessibility, Parker hydrophobicity, Karplus and Schulz flexibility prediction, and Chou and Fasman beta turn prediction methods at IEDB. SRVKNL epitope satisfies the thresholds of all prediction parameters in envelope protein. SRVKNL epitope was found to be antigenic, nontoxic, and nonallergic and conserved in all sequences of SARS-CoV-2 considered in this study. Thus, this epitope enables direct interactions with an immune receptor, which could be the putative vaccine candidates.

Since the immune response of T cell is a long-lasting response comparing with that of B cell, where the antigen can easily escape the antibody memory response [50] additionally, CD8+ and CD4+ T cell responses play a major role in antiviral immunity [51], designing of a vaccine against T-cell epitope is much more promising. Five MHC-I and MHC-II binding T-cell epitopes were predicted to interact with various HLA alleles. These epitopes are highly antigenic, nonallergic, and nontoxic in nature. These epitopes are found to be >99.9% conserved in all sequences of the SARS-CoV-2 considered in this study. This consistency of immunological features of epitopes indicates that these parameters fulfil all the criteria for further screening. The FLAFVVFLL and VLLFLAFVV epitopes were highly recommended as a candidate for the therapeutic peptide vaccine to interact with both MHC classes I and II. Also, the overlapping sequences of 9-mer CTL epitopes (16SVLLFLAFV24, 17VLLFLAFVV25, 18LLFLAFVVF26, and 26FLLVTLAIL34) from 16- to 34-amino acid region of E protein is forming an immunogenic domain. We found these CTL epitopes are overlapped with HTL epitopes. The overlapping between MHC class I and II T-cell epitopes suggested the possibility of antigen presentation to immune cells via both MHC class I and II pathways especially the overlapping sequences. All predicted HTL epitopes are IFN-*γ* inducing. Also, HTL epitopes ^31^LAILTALRL^39^ and ^57^YVYSRVKNL^65^ amino acid sequences overlapped with conformational B-cell epitopes. The 24-amino acid sequence from 16 to 39 of E protein could become a universal peptide-based vaccine against COVID-19 consisting of both B-cell and T-cell segments that may have the ability to enhance cell-mediated as well as humoral immunity. Further, docking study was performed with HTL and CTL epitopes to check interaction with MHC class I and II alleles. The binding affinity of these epitopes with MHC I and II alleles is very high with negative binding energy. These epitopes showed 91% coverage in the world, and the maximum population coverage was found in Europe (92%) and East Asia (90%).

*In silico* methods for vaccine development could be the alternative methods to conventional methods which are complex and time-consuming. Many bioinformatics-based approaches are used to design novel drugs [52, 53] and vaccines [54, 55] to curb this disease. *In silico* methods are effective, safe, and less time-consuming. However, till date, it is not clear which drug or medicine works better against the virus. It is a challenge to develop an effective vaccine and drug to combat this disease.

## 5. Conclusion

In this study, various bioinformatics tools are used to detect T- and B-cell epitopes from SARS-CoV-2 envelope protein and to assess their capability to recognize as antigens by the human immune system. One epitope, SRVKNL, has been proposed for an international therapeutic peptide vaccine for B cell. Also, the amino acid sequence from 16 to 39 of E protein may become a universal peptide-based vaccine against COVID-19. We recommend *in vitro* and *in vivo* validation for the efficacy and efficiency of these predicted candidate epitopes as a vaccine as well as to be used as a diagnostic screening test.

## Figures and Tables

**Table 1:** List of conformational B-cell epitopes of the E protein of SARS-CoV-2.

No.	Residues	Number of residues	Score
1	A: S60, A: R61, A: V62, A: K63, A: N64, A: L65	6	0.767
2	A: E8, A: T9, A: G10, A: T11, A: L12, A: I13, A: V14, A: S16	8	0.739
3	A: L51, A: V52, A: K53, A: S55, A: F56, A: Y59	6	0.658
4	A: A32, A: I33, A: T35, A: A36, A: L37, A: R38, A: L39, A: C40, A: A41, A: Y42	10	0.61

**Table 2:** List of CTL epitopes that have good combined score and are antigenic, nonallergic, and immunogenic, and bind with an affinity IC50 value of less than 200 with the MHC-I alleles.

Epitopes	Position in sequence	Combined score	Interaction of MHC-I allele with an affinity IC50 value of <200	Immunogenicity	Antigenic	Allergen	Toxic	Population coverage (%)	Binding score (kcal/mol) of epitopes with MHC-I
FLAFVVFLL	20	1.44	HLA-A*∗*02:01 HLA-A*∗*02:06 HLA-B*∗*15:02 HLA-C*∗*03:03 HLA-A*∗*68:02	0.30	Yes	No	No	48.4	−61.8

FLLVTLAIL	26	1.42	HLA-C*∗*03:03 HLA-A*∗*02:01 HLA-B*∗*15:02 HLA-C*∗*14:02 HLA-A*∗*02:06	0.17	Yes	No	No	48.4	−57.2

LLFLAFVVF	18	1.25	HLA-B*∗*15:01 HLA-A*∗*32:01 HLA-C*∗*12:03 HLA-C*∗*14:02 HLA-B*∗*15:02 HLA-C*∗*03:03	0.23	Yes	No	No	32.5	−43.31

VLLFLAFVV	17	1.12	HLA-A*∗*02:01 HLA-A*∗*02:06 HLA-C*∗*12:03 HLA-C*∗*14:02	0.26	Yes	No	No	48.4	−80.3

SVLLFLAFV	16	1.05	HLA-A*∗*02:06 HLA-C*∗*12:03 HLA-A*∗*02:01 HLA-A*∗*68:02	0.19	Yes	No	No	32.5	−60.31

**Table 3:** List of HTL epitopes that are antigenic and nonallergic and bind with an affinity IC50 value of less than 200 nm with MHC-II alleles.

Epitope	Position in sequence	Interaction of MHC-II alleles having IC50 value <200 nm	Antigenic	Allergen	Toxic	IFN-*γ* epitope prediction	Population coverage (%)	Binding score (kcal/mol) of epitopes with MHC-II
VLLFLAFVV	17	HLA-DRB1*∗*15:01 HLA-DRB1*∗*12:01 HLA-DRB1*∗*04:05 HLA-DRB1*∗*04:04 HLA-DRB1*∗*01:01 HLA-DRB5*∗*01:01 HLA-DRB1*∗*07:01	Yes	No	No	Positive	53	−90.4

FLAFVVFLL	20	HLA-DRB1*∗*04:05 HLA-DRB1*∗*04:04 HLA-DRB1*∗*15:01 HLA-DRB1*∗*04:01 HLA-DRB1*∗*01:01 HLA-DRB1*∗*07:01 HLA-DRB1*∗*04:04 HLA-DRB1*∗*09:01 HLA-DRB5*∗*01:01	Yes	No	No	Positive	62	−80.05

LLVTLAILT	27	HLA-DRB1*∗*01:01 HLA-DRB1*∗*15:01 HLA-DRB1*∗*07:01 HLA-DRB1*∗*04:04 HLA-DRB1*∗*07:01 HLA-DRB1*∗*04:05 HLA-DRB1*∗*07:01 HLA-DRB1*∗*15:01 HLA-DRB1*∗*11:01	Yes	No	No	Positive	57	−51.01

LAILTALRL	31	HLA-DRB1*∗*01:01 HLA-DRB1*∗*15:01 HLA-DRB1*∗*07:01 HLA-DRB1*∗*04:04 HLA-DRB1*∗*04:05 HLA-DRB1*∗*11:01	Yes	No	No	Positive	57	−64.52

YVYSRVKNL	57	HLA-DRB1*∗*04:01 HLA-DRB1*∗*04:05 HLA-DRB1*∗*07:01 HLA-DRB1*∗*11:01 HLA-DRB5*∗*01:01 HLA-DRB1*∗*01:01	Yes	No	No	Positive	48	−55.9

## Data Availability

The data used to support the findings of this study are available as a part of the article, and a supplementary file is associated with an article.

## Supplementary Materials

Supplementary Materials All sequences of the SARS-CoV-2 considered in this study are given.
